# The data do not support the existence of an ‘Old Boy network’ in science. Some critical comments on a study by Massen et al.

**DOI:** 10.1038/s41598-020-69278-3

**Published:** 2020-08-14

**Authors:** Freek Van de Velde, Benedikt Heller

**Affiliations:** 1 Department of Linguistics, Research Unit QLVL, KU Leuven, Blijde Inkomststraat 21/3308, 3000 Leuven, Belgium; 2 Department of English, Justus Liebig University Giessen, Otto-Behaghel-Strasse 10 B, 35394 Giessen, Germany

**Keywords:** Human behaviour, Biological anthropology

**arising from**: J.J.M. Massen et al.; *Scientific Reports* 10.1038/s41598-017-13491-0 (2017).

## Introduction

In “Sharing of science is most likely among male scientists”^1^ Massen et al. report on an intriguing study. They were interested in the positive response rate of academics whom they asked to share either a paper or a dataset. Controlling for a number of variables, the authors conclude that male scientists are more likely to share but mainly with other male scientists, which they putatively ascribe to an “Old Boy network.” However, upon close inspection, the data do not warrant their conclusions.

To begin with, there is a problem with the interaction term on which the authors base their main claim: in our view, it is both misinterpreted and misspecified. Their claim (i.e., “Sharing of science is most likely among male scientists”) involves the effects of Sex of Requester and Sex of Participant. In regression terms, this translates into a two-way interaction (i.e., Sex of Requester: Sex of Participant). Massen et al., however, base their claim on a three-way interaction of the above with Condition (i.e., paper vs. dataset requests). This is questionable because a model with just a two-way interaction and a control for condition is not only more parsimonious and conceptually closer to what they set out to show but also yields a superior Akaike Information Criterion (AIC) value (i.e., a lower one). AIC is the benchmark used by the authors themselves, and it indicates that the three-way interaction is in fact superfluous. Now crucially, in the parsimonious model, the interaction between Sex of requester and Sex of Participant is not significant (*p* = 0.061).

Beyond the specification of this crucial interaction term, we take issue with the authors’ modelling procedure. Upon request, they reported that their model does not include lower-order terms (in particular, the aforementioned crucial interaction term Sex of Requester: Sex of Participant). There are two problems with this. First, this approach violates the ‘Principle of marginality’^2,3^, constituting an “arbitrary imposition on the model.”^4^ In principle, there can be reasons to deviate from this principle under specific (and very rare) circumstances^5^, but there should be an independent motivation for doing so. No such motivation was given, and we cannot see a reason ourselves. Second, omitting the crucial lower-order term sex of requester: sex of participant performs a different test than implied. The three-way interaction without the two-way interaction contrasts male-male paper requests with every other combination of the three predictors. Since this also includes male-male data requests, the statistical meaning of the interaction term is obfuscated. (As an aside: the interaction in the regression pits everything against male-male sharing in the low-cost condition, i.e., paper requests, and not, as implied in the results section, against male-male sharing in the high-cost condition, i.e., data requests).

In short, considering both the misinterpretation and the misspecification of the crucial interaction term, no clear connection between the authors’ analysis and their claims remains, in our view.

Next, we noticed some problems in the regression model in the supplementary materials and failed to replicate the analysis following the same protocol. The authors use a backward model selection procedure based on AIC. Although one could take issue with such a procedure in hypothesis-driven research (as opposed to exploratory research), we did follow the same procedure, and arrived at different conclusions. Starting from a model that contains all effects that the article lists as significant and using an AIC-based backwards selection procedure, we obtain a model which does contain the variables condition, h-index, sex of requester, and sex of participant, as well the interaction sex of requester: sex of participant, but of these regressors, only one is significant, viz. Condition (paper vs. dataset). This result is also confirmed by a (non-parametric) conditional inference tree analysis (see Supplementary Information, https://github.com/FreekVandeVelde/Old-Boy-Network-in-Science). The tree only selects one variable: paper vs. dataset. In sum, we do not see how one would arrive at the model the authors report; if we include lower-order terms (as is standard procedure), *none* of the explanatory variables that Massen et al. retain in their final model reach statistical significance at *p* < 0*.*05. We do not mean to defend the *p* < 0*.*05 threshold uncritically, but given the multiple testing inherent in backwards model selection, we believe it to be a fairly liberal maximum.

In order to assess the danger that lies in the authors’ procedure numerically, we ran a simulation that reveals how often the focal interaction term (i.e., sex of the requester by sex of the participant) is to be expected to turn up as significant (i.e., *p* < 0.05) given random data. In other words, the simulation was designed to reflect the interaction’s true false positive rate. In each iteration, the simulation generated random data (for binary predictors—i.e., sex of the requester, sex of the participant, status of the requester, condition and response—values were chosen randomly with p = 0.5; values of h-index where randomly sampled from the original dataset to mimic their distribution). This simulation was run 10,000 times. Results indicate an alarming false positive rate of the focal interaction of close to 30%, which echoes Ioannidis’s and Nuzzo’s concerns that such a research design is liable to type 1 errors.^6,7^ Considering that this interaction term does not even reach statistical significance in Massen et al.’s study if the model is properly specified (i.e., including lower-order terms), we question the validity of their most central finding. If we were to follow standard procedure (see^8^, *inter alia*), regression analysis attributes the variance in the data to the main effect of Sex of Participant, i.e., remains limited to a higher generosity of male respondents.

In sum, taking into account (1) the misinterpretation and misspecification of the crucial interaction term, (2) the failure to reach statistical significance with a valid model formula, and (3) the 30% expected false positive rate following Massen et al.’s model selection procedure in a simulation with random data, we doubt the validity of the authors’ central claim that “Sharing of science is most likely among male scientists.” We acknowledge a trend that is visible in Massen et al.’s Fig. 1, but under multivariate control, this trend breaks down. It is very likely to be a spurious artefact of the procedure applied.

With a difficult topic such as a pro-male gender bias in academia, leading journals, such as *Scientific Reports*, have a responsibility to maintain high standards in methodology. It has been credibly suggested by Duarte and his colleagues^9^ and subsequent commentaries by Baumeister, by Funder, and by Pinker that research relating to these issues is often not discussed with the much needed scientific objectivity (see also^10,11^). Though there are certainly studies that purport to show that women are at a disadvantage in academia, a number of carefully argued recent studies have shown that the underrepresentation of women in some academic fields is not due to an Old Boy network, where males favor their own in hiring.^12,13^ The final verdict has not fallen in this hotly debated issue, and much remains unclear. Awaiting new research, we ought not abandon the null hypothesis: by Occam’s razor, the Old Boy network remains a mirage in the dataset at hand.

## Data Availability

10.5281/zenodo.3891563. https://github.com/FreekVandeVelde/Old-Boy-Network-in-Science.
